# Multi‑institutional development and validation of a nomogram to predict prognosis of early-onset gastric cancer patients

**DOI:** 10.3389/fimmu.2022.1007176

**Published:** 2022-09-06

**Authors:** Hongda Liu, Zequn Li, Qun Zhang, Qingya Li, Hao Zhong, Yawen Wang, Hui Yang, Hui Li, Xiao Wang, Kangshuai Li, Dehai Wang, Xiangrong Kong, Zhongyuan He, Weizhi Wang, Linjun Wang, Diancai Zhang, Hao Xu, Li Yang, Yuxin Chen, Yanbing Zhou, Zekuan Xu

**Affiliations:** ^1^ Department of General Surgery, The First Affiliated Hospital of Nanjing Medical University, Nanjing, China; ^2^ Department of Gastrointestinal Surgery, The Affiliated Hospital of Qingdao University, Qingdao, China; ^3^ Department of Respiratory Medicine, The First Affiliated Hospital of Nanjing Medical University, Nanjing, China; ^4^ Department of General Surgery, Qilu Hospital of Shandong University, Jinan, China; ^5^ Department of General Surgery, The First Affiliated Hospital of Shandong First Medical University and Shandong Qianfoshan Hospital, Jinan, China; ^6^ Department of Pathology, The Second Hospital Affiliated to Shandong University, Jinan, China; ^7^ Department of Plastic Surgery, The Second Hospital Affiliated to Shandong University, Jinan, China; ^8^ Department of Gastrointestinal Surgery, The Second Hospital Affiliated to Shandong University, Jinan, China; ^9^ Qingdao Urban Planning and Design Research Institute, Qingdao, China

**Keywords:** early-onset gastric cancer (EOGC), gastrectomy, nomogram, SEER, survival calculator

## Abstract

**Background:**

Early-onset gastric cancer (EOGC, ≤45 years old) is characterized with increasing incidence and more malignant phenotypes compared with late-onset gastric cancer, which exhibits remarkable immune cell infiltration and is potential immunotherapeutic population. Till now, restricted survival information of EOGC is available due to limited case numbers. This study established a novel nomogram to help evaluate cancer-specific survival (CSS) of EOGC patients who underwent gastrectomy, and may provide evidence for predicting patients’ survival.

**Methods:**

We retrospectively enrolled a cohort containing 555 EOGC cases from five independent medical centers in China, among which 388 cases were randomly selected into a training set while the other 167 cases were assigned into the internal validation set. Asian or Pacific Islander (API) patients diagnosed with EOGC during 1975-2016 were retrieved from the SEER database (n=299) and utilized as the external validation cohort. Univariate and multivariate analyses were conducted to test prognostic significances of clinicopathological factors in the training set. Accordingly, two survival nomogram models were established and compared by concordance index (C-index), calibration curve, receiver operating characteristics (ROC) curves and decision curve analyses (DCA).

**Results:**

The 5-year CSS rate of training cohort was 61.3% with a median survival time as 97.2 months. High consistency was observed on calibration curves in all three cohorts. Preferred nomogram was selected due to its better performance on ROC and DCA results. Accordingly, a novel predicative risk model was introduced to better stratify high-risk EOGC patients with low-risk patients. In brief, the 5-year CSS rates for low-risk groups were 92.9% in training set, 83.1% in internal validation set, 89.9% in combined NQSQS cohort, and 85.3% in SEER-API cohort. In contrast, the 5-year CSS rates decreased to 38.5%, 44.3%, 40.5%, and 36.9% in the high-risk groups of the four cohorts above, respectively. The significant survival difference between high-risk group (HRG) and low-risk group (LRG) indicated the precise accuracy of our risk model. Furthermore, the risk model was validated in patients with different TNM stages, respectively. Finally, an EOGC web-based survival calculator was established with public access, which can help predict prognosis.

**Conclusions:**

Our data provided a precise nomogram on predicting CSS of EOGC patients with potential clinical applicability.

## Introduction

Gastric cancer accounts for 5.6% among all cancer cases, ranking 5^th^ on incidence and 4^th^ on mortality rate worldwide (1). According to the Surveillance, Epidemiology and End Results (SEER) database, a significantly increased incidence was observed in younger populations although the rate for entire population declined in the past decades (2). Therefore, early-onset gastric cancer (EOGC, ≤ 45 years old) is attracting more and more attentions on both clinical aspects and mechanisms. Accounting for 2.7-10% of all gastric cancers, EOGC possesses different clinicopathological and molecular-genetic characteristics comparing with late-onset gastric cancers (LOGC, > 45 years old), including diffuse lesions, poorer differentiation grade and hereditary genetic alterations (3). As a result, EOGC is more frequent to demonstrate advanced stages at the time of diagnosis with low resectability, thus possessing a median overall survival time as short as 11.7 months (4). It has been well-acknowledged that patients may show completely different outcomes even in the same TNM stage due to high heterogenicity, indicating that TNM stage can be further improved for specific populations. Therefore, a more specific risk model is essential for predicting clinical outcomes and guiding the management of EOGC patients.

Comparing with American and European countries, Asian and Pacific Islander (API) suffered a higher burden on gastric cancer. For example, there are 138, 470 new cases in Japan and 478, 508 new cases in China, comprising more than half of all gastric cancers worldwide in 2020 (1). Interestingly, although with higher incidence, API ethnicity exhibits distinct characteristics and better outcomes of gastric cancer after surgical intervention compared with non-API (5–7). Till now, there is no predicative model for prognostic evaluation of API EOGC cases.

Here we collected 555 EOGC patients from five independent medical centers in China, which was randomly divided into training cohort and internal validation cohort. Meanwhile, API EOGC patients were selected from the SEER database (SEER-API cohort, n=299) and utilized as the external validation cohort. After establishing the nomogram to predict cancer specific survival (CSS) based on the training set, the model performance was evaluated by both internal validation cohort and external cohort. Furthermore, we confirmed the potential clinical application of our nomogram on predicting CSS by distinguishing patients into low- and high-risk stratifications. Finally, a website for survival prediction was provided for EOGC patients.

## Methods

### Study design and cohort selection

Although refers to young cases, definition of EOGC remains controversial regarding the cut-off age (8–10). Here we adopted the definition as diagnosed at the age of 45 years or younger (8).

A retrospective cohort of EOGC (n=555) was collected from five medical centers in China, including Nanjing Medical University First Affiliated Hospital (2010.2-2017.8), Qingdao University Affiliated Hospital (2014.8-2017.12), Shandong University Affiliated Qilu Hospital (2013.1-2018.12), Qianfoshan Hospital (2013.11-2019.5), and Second Affiliated Hospital of Shandong University (2013.7-2019.6). This cohort was named as NQSQS cohort and were further randomly divided into a training set (n=388) and an internal validation set (n=167). Similarly, a retrospective cohort of EOGC in API population was retrieved from SEER database during 1975 to 2016, which was named as SEER-API cohort (n=299) and utilized for external validation.

**Figure 1:**
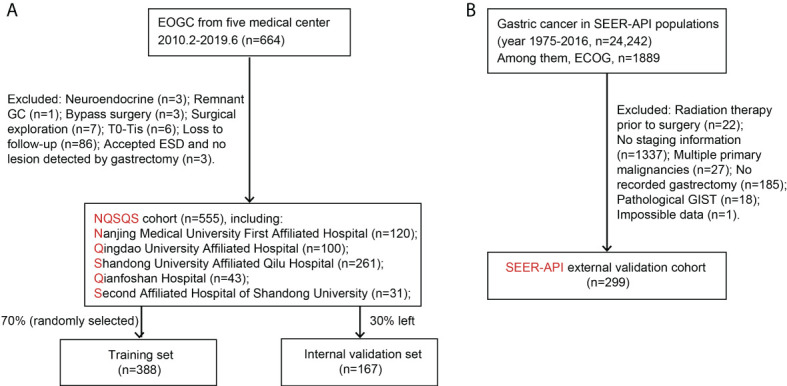
Patients’ enrollment and exclusion flow chart. **(A)** The NQSQS cohort included 555 EOGC patients from five medical centers in China, which was further divided into training set (n=388) and internal validation set (n=167). **(B)** The SEER-API cohort included 299 Asian or Pacific Islander (API) EOGC patients selected from SEER dataset.

For both NQSQS cohort and SEER-API cohort, the inclusion criteria were: (i) patients who were histologically diagnosed as gastric adenocarcinoma at the age of 45 years or younger; (ii) with accurate API ethnicity; (iii) underwent gastrectomy therapy; (iv) without preoperative radiotherapy; (v) with intact TNM information; (vi) without multiple primary malignancies; (vii) with intact follow-up information regarding cancer-specific death. The patients’ inclusion and exclusion were summarized in **Figure 1**.

### Outcomes and variable definition

The endpoint was set as cancer-specific death and the follow-up time was defined as the period from the date of diagnosis to the date of cancer-specific death or the date of last follow-up. In the training cohort, the median CSS time was 97.2 months (ranging 0.5-131.3 months) with a 5-year CSS rate as 61.3%. In the internal validation cohort, the median CSS time was 73.2 months (ranging 0.6-127.1 months) with a 5-year CSS rate as 61.2%. In the SEER-API cohort, the median CSS time was 84.0 months (ranging 0.6-127.1 months) with a 5-year CSS rate as 51.8%.

The variables enrolled in this study included age at diagnosis, sex, anatomical tumor location, tumor size, histological type, differentiation grade, T stage, N stage, M stage, gastrectomy pattern, and number of dissected lymph nodes (LN). The age was modeled as a binary variable based on the median age (40 yrs) of training cohort. The tumor location was classified as gastric cardia, fundus, body, antrum, pylorus, overlapping or linitis plastica (11), or unspecified. Tumor size was evaluated based on the largest tumor diameter and grouped as ≤ 2.0 cm, between 2.0 and 5.0 cm, larger than 5.0 cm or unknown. Histological type was classified as adenocarcinoma (with ≤30% signet ring cells), signet ring cell carcinoma (with more than 30% signet ring cells), others (mucinous adenocarcinoma) or unknown (confirmed as adenocarcinoma by preoperative biopsy, but didn’t clearly described in the pathology reports after surgical resection). Differentiation grade was classified as undifferentiated, poor differentiated, moderate differentiated, well differentiated, or unknown. TNM stages were classified according to the AJCC classification system. The gastrectomy pattern was classified as total/subtotal gastrectomy, partial gastrectomy, or unspecified gastrectomy. The number of examined LN was sub-grouped into < 18 LNs or ≥18 LNs.

### Statistical analyses

Statistical analyses were performed using SPSS Software version 19.0 and package R version 3.3.0. The normality test was performed by Kolmogorov–Smirnov method. The survival time and survival rates were obtained by Kaplan-Meier method and log-rank test. Univariate and multivariate analyses were conducted by regression test to obtain hazard ratio (HR) and 95% confidence interval (CI). In the multivariate Cox regression model, all retrieved variables were adjusted to identify independent prognostic factors. X-tile software was used to determine the cut-off point to distinguish patients with high- or low-risk. Quantitative data were expressed using mean ± standard deviation (SD). A two-sided P<0.05 was considered statistically significant.

### Establishment and validation of nomograms

Two nomograms were established in this study using the data from training set. The model 1 was developed based on only three parameters including T stage, N stage, and M stage. The model 2 was developed based on all enrolled variables with P<0.10 in multivariate analysis. The concordance index (C-index) was calculated to evaluate the performance of two nomograms on all three cohorts, respectively. Calibration curves (1,000 bootstrap resamples) were also plotted to compare nomogram-predicted CSS and actual CSS of enrolled cohorts using a 45-degree line as an optimal model. Receiver operating characteristic (ROC) curves for the two nomograms were generated and compared based on the area under the curve (AUC). Meanwhile, the decision curve analysis (DCA) was utilized to assess the net benefit of the nomograms in a clinical context.

### Ethics

The Ethics Committee of The First Affiliated Hospital of Nanjing Medical University approved this retrospective study.

## Results

### Patients’ demographics

**Table 1** showed the demographic and clinicopathological characteristics of all three cohorts, including training set (n=388), internal validation set (n=167), and SEER-API external validation set (n=299). Interestingly, the ratio of male patients versus females ranged from 0.72-1.50 in the three cohorts without significant difference. Although a recent study reported 1.86 to 2.20 folds higher incidence in male patients for global gastric cancer (12), EOGC seems to show similar incidence in males and females. More than 80% cases in NQSQS cohort showed tumor localization in stomach body, antrum, or pylorus, while less than 10% cases showed cardia or fundus location. Similarly, the cardia or fundus location percentage was 8.7% in SEER-API cohort. Of note, more than 74% cases showed undifferentiated or poor differentiation grade in all the three cohorts, implying the high malignant behaviors of EOGC. Besides, approximately 60% EOGC patients showed positive lymph node metastasis in all the three cohorts. Although all the cases underwent R0 resection of primary gastric cancer lesion, there were 4.7% (26/555) patients with distant metastasis in NQSQS cohort, while up to 22.7% (68/299) M1 cases in SEER-API cohort. In all the three cohorts, the percentage of total or subtotal resection of stomach was less than 30%. Of note, the median number of dissected lymph nodes was 25 (1-65) in NQSQS cohort, while was only 18 (0-95) in SEER-API cohort. Another significant difference is the acceptance of chemotherapy treatment. There were 74.2% cases accepted chemotherapy treatment in SEER-API cohort, while only less than 30% cases accepted postoperative chemotherapy in NQSQS cohort.

**Table 1:** Demographic and clinicopathologic characteristics of the training and validation cohorts.

Variables	Training cohort(n=388)		Internal validation cohort (n=167)		External validation cohort (n=299)	
	**Case No. (n)**	**%**	**Case No. (n)**	**%**	**Case No. (n)**	**%**
**Age**						
< 40 yrs	184	47.4%	80	47.9%	136	45.50%
≥ 40 yrs	204	52.6%	87	52.1%	163	54.50%
**Sex**						
Female	182	46.9%	67	40.1%	173	57.90%
Male	206	53.1%	100	59.9%	126	42.10%
**Tumor location**						
Cardia/Fundus	36	9.3%	16	9.6%	26	8.70%
Body/antrum/pylorus	314	80.9%	138	82.6%	146	48.80%
Unspecified	19	4.9%	11	6.6%	90	30.10%
Overlapping/Linitis plastica	19	4.9%	2	1.2%	37	12.40%
**Tumor size**						
≤ 2.0 cm	97	25.0%	38	22.8%	55	18.40%
2.0-5.0 cm	191	49.2%	95	56.9%	102	34.10%
> 5.0 cm or unknown	100	25.8%	34	20.4%	142	47.50%
**Histological type**						
Adenocarcinoma	230	59.3%	112	67.1%	153	51.20%
Signet ring cell carcinoma	141	36.3%	47	28.1%	126	42.10%
Others or unknown	17	4.4%	8	4.8%	20	6.70%
**Differentiation grade**						
Undifferentiated/poor	291	75.0%	124	74.3%	258	86.30%
Moderate/well/unknown	97	25.0%	43	25.7%	41	13.70%
**T stage**						
T1-T2	139	35.8%	65	38.9%	135	45.20%
T3	118	30.4%	52	31.1%	92	30.80%
T4	131	33.8%	50	29.9%	72	24.10%
**N stage**						
N0	159	41.0%	60	35.9%	111	37.10%
N1	61	15.7%	30	18.0%	43	14.40%
N2	60	15.5%	30	18.0%	47	15.70%
N3	108	27.8%	47	28.1%	98	32.80%
**M stage**						
M0	371	95.6%	158	94.6%	231	77.30%
M1	17	4.4%	9	5.4%	68	22.70%
**Gastrectomy pattern**						
Total or subtotal	95	24.5%	44	26.3%	89	29.80%
Partial	283	72.9%	122	73.1%	201	67.20%
Gastrectomy, NOS	10	2.6%	1	0.6%	9	3.00%
**Dissected lymph nodes**						
≤ 18 or unknown	66	17.0%	32	19.2%	156	52.20%
> 18	322	83.0%	135	80.8%	143	47.80%
**Chemotherapy**						
Untreated or unknown	287	74.0%	114	68.3%	77	25.80%
Accepted	101	26.0%	53	31.7%	222	74.20%
**Median follow-up months**	39.3		44.0		32.0	
**No. of deaths (%)**	144 (37.1%)		66 (39.5%)		139 (46.5%)	
**Median CSS months (range)**	97.2 (0.5-131.3)		73.2 (0.6-127.1)		84.0 (3.0-152.0)	
**5-year CSS rate**	61.3%		61.2%		51.8%	

### Univariate and multivariate analyses of training cohort

Univariate analysis was firstly conducted using the Kaplan-Meier method based on each enrolled variable (**Table 2**). Accordingly, EOGC patients with stomach body/antrum/pylorus tumor location exhibited significantly better prognosis than those with cardia/fundus location (mean CSS time 85.8 ± 4.2 vs. 47.1 ± 5.2 months). Meanwhile, the 5-year CSS rate was only 7.0% in patients with overlapping tumor location or linitis plastica. Consistently, patients accepted partial gastrectomy showed longer CSS time than those underwent total or subtotal gastrectomy (91.4 ± 3.7 vs. 53.6 ± 5.5 months). As expected, patients with larger tumor size exhibited poorer survival (P<0.001). Other significant unfavorable prognostic factors included poorer differentiation grade, advanced T stage, N stage, M stage, and absent of chemotherapy (all P<0.05). Of note, although the number of dissected lymph nodes showed no statistical significance, the median CSS time was about 10 months longer in those with more than 18 dissected lymph nodes, highlighting the benefit of D2 lymphadenectomy.

**Table 2:** Univariate and multivariate Cox regression analyses of factors associated with CSS in the training set.

Variables	Univariate						Multivariate		
	5-y CSS(%)	Mean ± S.D(months)	Median(months)	HR	95% CI	P value ^a^	HR	95% CI	P value ^b^
**Age**						0.190			0.940
< 40 yrs	57.2%	74.9 ± 5.3	83.2	Reference			Reference		
≥ 40 yrs	64.9%	75.0 ± 3.8	102.1	0.804	0.579-1.115		0.986	0.691-1.407	
**Sex**						0.366			0.391
Female	58.0%	74.2 ± 4.3	102.1	Reference			Reference		
Male	64.4%	78.8 ± 6.2	97.2	0.860	0.620-1.193		1.174	0.814-1.694	
**Tumor location**						<0.001*			<0.001*
Cardia/Fundus	38.5%	47.1 ± 5.2	45.2	Reference			Reference		
Body/antrum/pylorus	68.5%	85.8 ± 4.2	102.1	0.487	0.301-0.790		0.580	0.343-0.982	
Unspecified	47.8%	56.0 ± 7.8	48.9	0.727	0.320-1.651		1.521	0.608-3.804	
Overlapping/Linitis plastica	7.0%	19.3 ± 3.7	15.3	2.792	1.457-5.351		1.913	0.959-3.818	
**Tumor size**						<0.001*			0.036*
≤ 2.0 cm	91.5%	118.3 ± 4.2	–	Reference			Reference		
2.0-5.0 cm	56.5%	72.6 ± 4.2	97.2	5.319	2.665-10.614		2.202	1.033-4.695	
> 5.0 cm or unknown	41.4%	46.9 ± 3.9	38.7	9.127	4.512-18.461		2.794	1.266-6.166	
**Histological type**						0.082			0.234
Adenocarcinoma	56.9%	71.2 ± 3.9	82.2	Reference			Reference		
Signet ring cell carcinoma	67.1%	71.2 ± 3.3	97.2	0.690	0.482-0.987		0.716	0.487-1.052	
Others or unknown	73.1%	94.7 ± 13.2	–	0.591	0.240-1.453		0.855	0.318-2.295	
**Differentiation grade**						0.010*			0.022*
Undifferentiated/poor	57.5%	74.1 ± 4.4	86.9	Reference			Reference		
Moderate/well/unknown	72.9%	84.7 ± 4.7	–	0.577	0.377-0.882		0.567	0.349-0.923	
**T stage**						<0.001*			0.095
T1-T2	88.5%	108.0 ± 7.4	–	Reference			Reference		
T3	52.8%	65.3 ± 4.7	86.9	4.772	2.724-8.362		1.793	0.971-3.309	
T4	42.0%	52.5 ± 4.3	39.6	6.663	3.883-11.434		1.946	1.064-3.559	
**N stage**						<0.001*			<0.001*
N0	88.0%	111.0 ± 5.1	–	Reference			Reference		
N1	71.8%	85.2 ± 7.0	–	2.596	1.361-4.953		1.830	0.940-3.562	
N2	44.1%	62.0 ± 6.1	45.9	5.279	2.979-9.353		3.149	1.688-5.873	
N3	29.8%	40.4 ± 3.8	26.3	8.963	5.410-14.849		5.004	2.884-8.683	
**M stage**						<0.001*			<0.001*
M0	63.3%	81.5 ± 3.9	97.2	Reference			Reference		
M1	22.1%	23.3 ± 5.3	13.6	4.171	2.342-7.430		3.746	1.999-7.021	
**Gastrectomy pattern**						<0.001*			0.213
Total or subtotal	40.9%	53.6 ± 5.5	39.6	Reference			Reference		
Partial	68.2%	91.4 ± 3.7	–	0.426	0.303-0.600		0.765	0.516-1.132	
Gastrectomy, NOS	60.0%	63.0 ± 14.9	97.2	0.744	0.297-1.866		1.435	0.540-3.815	
**Dissected lymph nodes**						0.905			0.086
≤ 18 or unknown	60.7%	80.2 ± 7.5	86.9	Reference			Reference		
> 18	61.5%	71.0 ± 3.0	97.2	0.974	0.635-1.496		0.676	0.432-1.057	
**Chemotherapy**						0.003*			0.012*
Untreated or unknown	56.9%	73.6 ± 4.1	82.2	Reference			Reference		
Accepted	73.5%	87.8 ± 4.4	–	0.524	0.340-0.807		0.542	0.337-0.872	

a. P value was calculated by log-rank test.

b. P value was calculated by Cox-regression test.* indicates P<0.05.

In addition, we performed multivariate analysis to further identify potential risk factors for EOGC patients (**Table 2**). As a result, independent risk factors included tumor location (P<0.001), tumor size (P=0.036), differentiation grade (P=0.022), N stage (P<0.001), M stage (P<0.001), and chemotherapy (P=0.012).

### Variable selection and nomogram development

**Figure 2:**
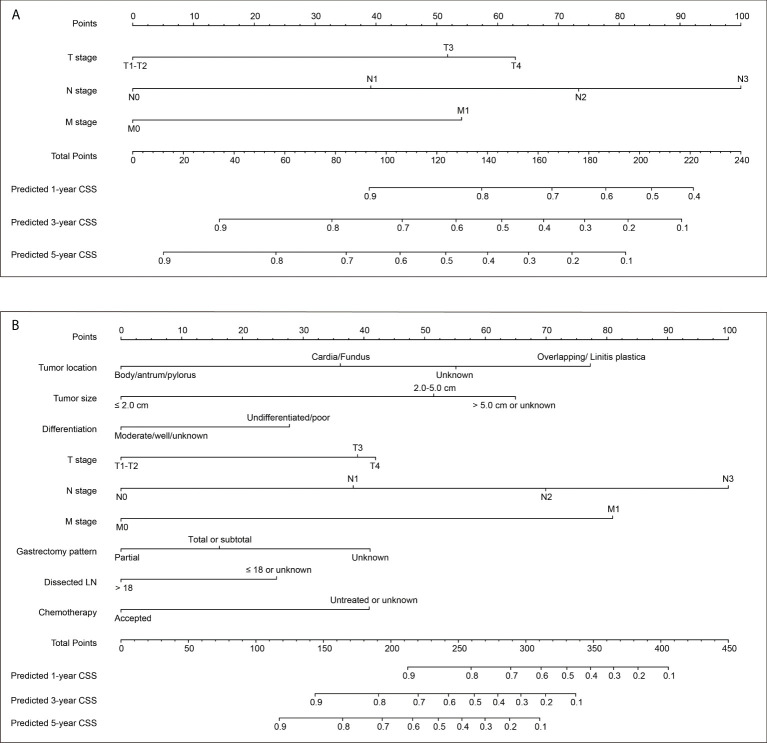
Two nomograms to predict 1-, 3− and 5−year cancer-specific survival (CSS) rates of EOGC. **(A)** Model 1 nomogram was established based on tumor T stage, N stage, and M stage. **(B)** Model 2 nomogram was established based on tumor location, tumor size, differentiation grade, T stage, N stage, M stage, gastrectomy pattern, number of dissected lymph nodes, and postoperative chemotherapy.

Here we introduced two nomograms. The model 1 was a simpler one, which was generated according to the three most conventional and critical variables, including T stage, N stage, and M stage (**Figure 2A**). The model 2 was a relatively complicated one (**Figure 2B**), which included six statistically significant variables (tumor location, tumor size, differentiation grade, N stage, M stage, and chemotherapy) according to the multivariate analysis of training set. Besides, T stage, gastrectomy pattern, and number of dissected lymph nodes were also included in model 2 due to their clinical significance although without statistically significance in the training set.

Both the two nomograms were used to predict 1-, 3− and 5−year CSS rates using hazard ratios from the Cox multivariate results from the training set (n=388) as mentioned above. Briefly, each subtype of enrolled covariates was assigned a point. Therefore, by adding the total points together and locating it on the bottom scale, we can calculate the probability of 1-, 3− and 5−year CSS. Based on the training set, the C-index of model 1 was 0.769 with 95% CI 0.736-0.802, while the C-index of model 2 was 0.798 with 95% CI 0.763-0.833 (**Table S1**). The C-index of model 1 was 0.732 and 0.803 for internal validation cohort and SEER-API cohort, respectively. The C-index of model 2 was 0.771 and 0.826 for internal validation cohort and SEER-API cohort, respectively.

### Validation and comparation of two nomograms

**Figure 3:**
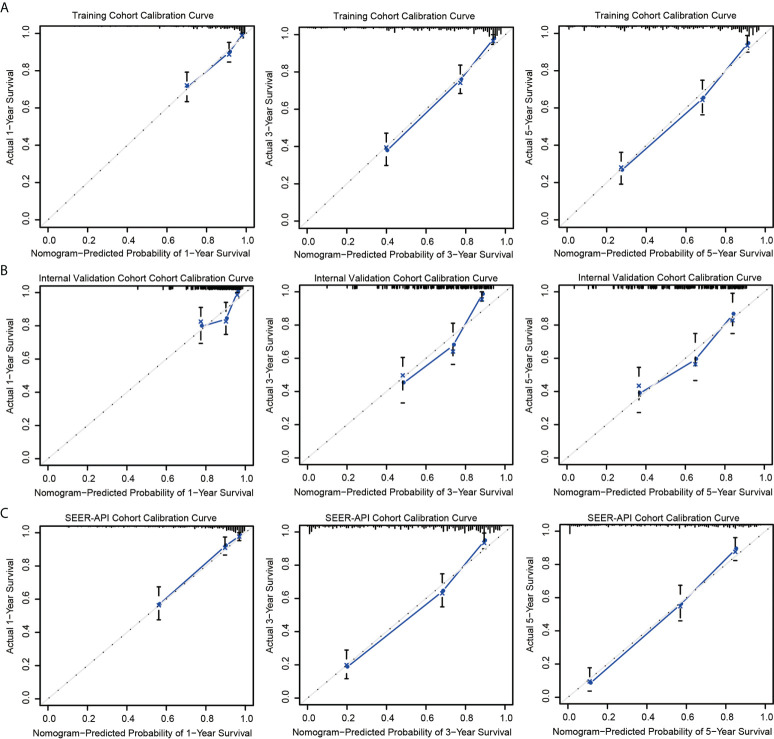
Calibration curves of model 2 nomogram. Calibration curves of the nomogram in the training set **(A)**, internal validation set **(B)**, and SEER-API external validation set **(C)** were plotted based on 1-, 3-, and 5-year CSS, respectively. The X-axis represents the model−predicted survival, and the Y−axis represents actual survival. The bar represents 95% CI measured by Kaplan–Meier analysis, and the dotted line represents the ideal reference line.

The predictive accuracies of two nomograms were next validated in training cohort, internal validation cohort, and SEER-API cohort, respectively. As a result, the calibration plots performed well in all the three cohorts (**Figure 3**, **Figure S1**).

**Figure 4:**
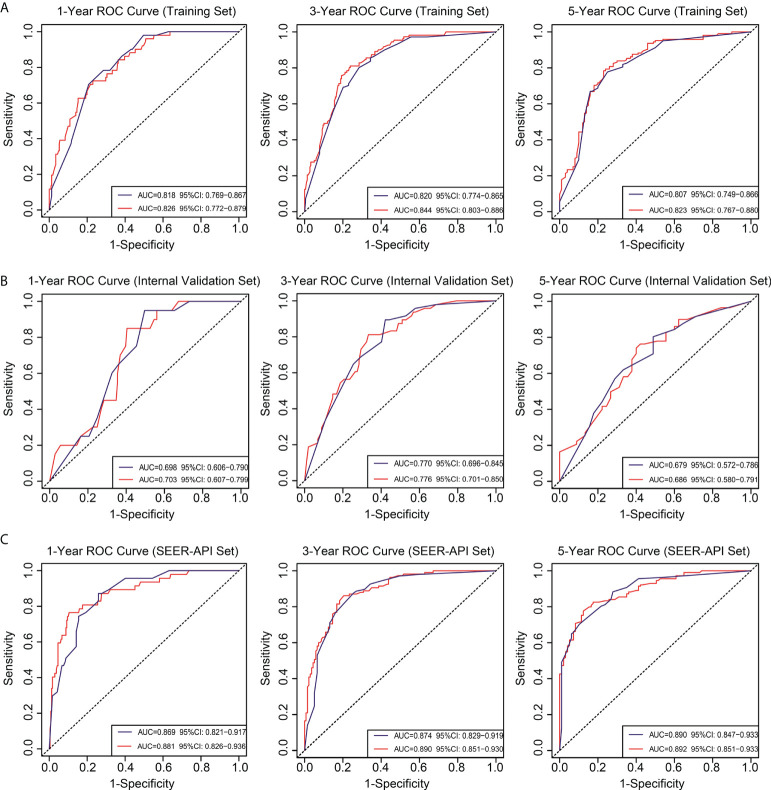
Receiver operating characteristic (ROC) curves for the two nomograms. Based on 1-, 3-, and 5-year CSS, the ROC curves for the nomograms were plotted in the training set **(A)**, internal validation set **(B)**, and SEER-API external validation set **(C)**, respectively. The blue line represents model 1, and the red line represents model 2.

**Figure 5:**
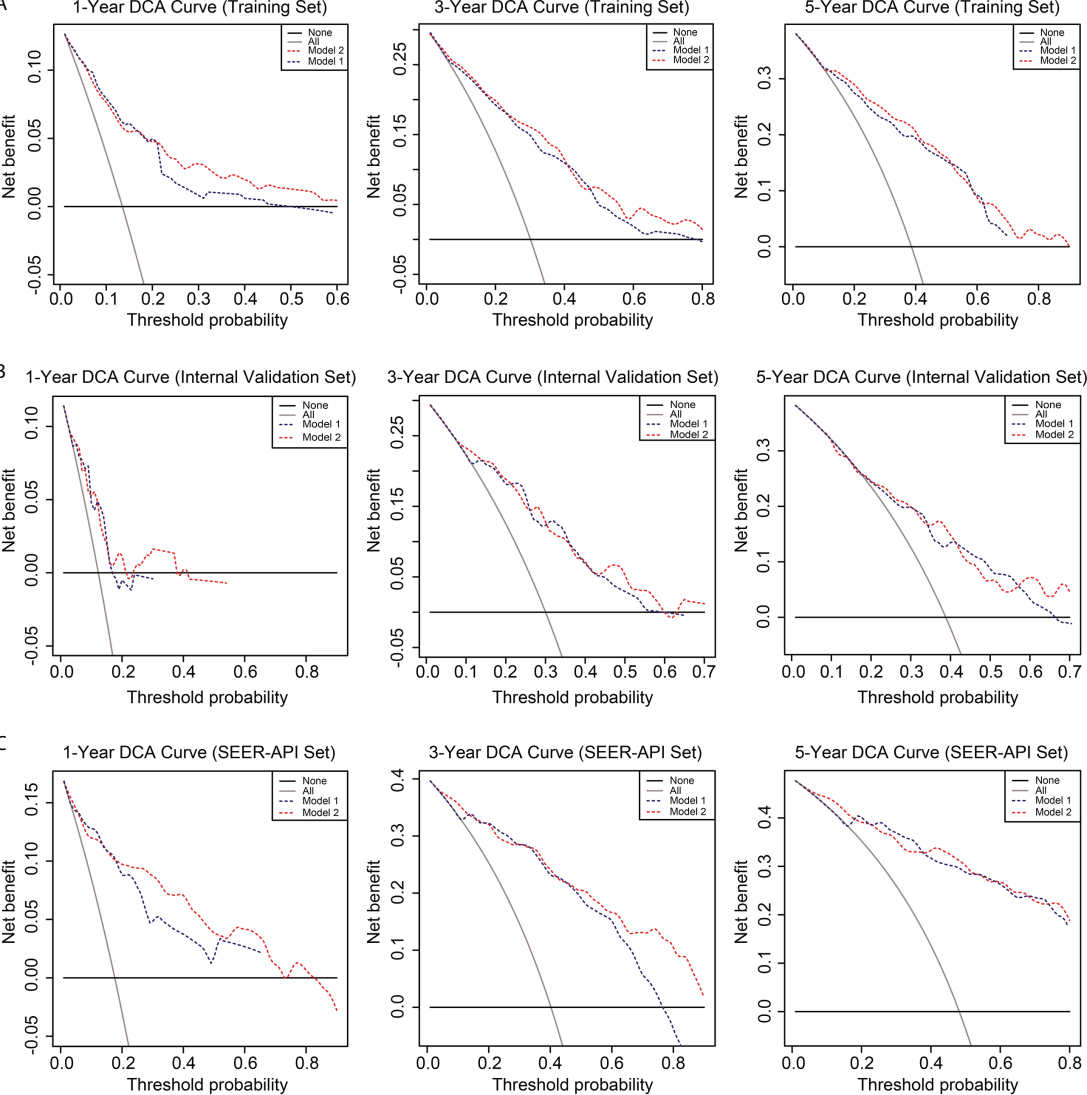
Decision curve analysis (DCA) of the two nomograms. The DCA curves of two nomograms in the training set **(A)**, internal validation set **(B)**, and SEER-API external validation set **(C)** were plotted based on 1-, 3-, and 5-year CSS, respectively. The blue line represents model 1, and the red line represents model 2.

To further compare the two nomograms, ROC curves and DCA curves were plotted. Accordingly, model 2 showed higher AUC in all the ROC curves from three cohorts (**Figure 4**), indicating its better prognostic accuracy compared with model 1. Consistently, DCA curves also revealed a better performance of model 2 (**Figure 5**). However, because there are several multicollinearities in model 2 factors, the DCA curves in model 2 somehow shows similarity with those in model 1. Nevertheless, model 2 was selected for further analyses in this study.

### Risk model establishment based on the nomogram

Each patient in the training set (n=388) was given a risk score according to the nomogram model 2 (**Figure 2B**), ranging 42-409 scores. The cut off score was determined by X-tile software based on the survival data, which aimed to obtain the smallest P value of the χ2 log-rank test thus determine the threshold to distinguish high- or low-risk patients (**Figure S2**). Accordingly, the cut off value of risk score was set as 188. Therefore, patients in the training set with risk scores ≤188 were grouped as low-risk group (n=166), while those with risk scores >188 were grouped as high-risk group (n=222). Similarly, 75 cases in the internal validation group were classified as low-risk patients, while the other 92 cases were defined with high-risk. As for the SEER-API external validation cohort, there were 91 low-risk patients and 208 high-risk patients.

**Figure 6:**
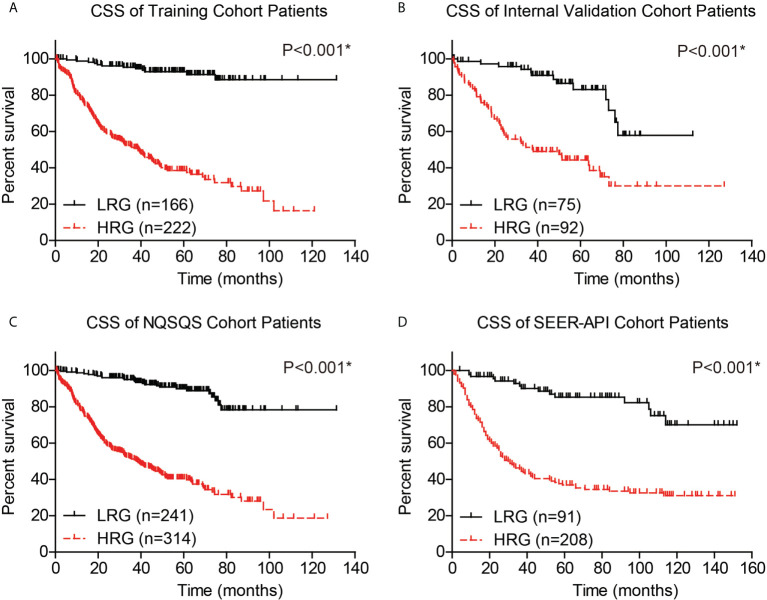
Cancer-specific survival (CSS) analyses of EOGC patients based on the risk model. Based on the risk model, EOGC patients were divided into high-risk group (HRG) and low-risk group (LRG), then the CSS curves were plotted in the training set **(A)**, internal validation set **(B)**, combined NQSQS cohort **(C)**, and SEER-API external validation set **(D)**, respectively. *indicates P<0.05 by log-rank test.

The performance of our risk model was then evaluated by plotting the CSS survival curves of training cohort, internal validation cohort, combined NQSQS cohort, and SEER-API cohort, respectively (**Figure 6**). In brief, the 5-year CSS rates for low-risk groups were 92.9% in training set, 83.1% in internal validation set, 89.9% in combined NQSQS cohort, and 85.3% in SEER-API cohort. In contrast, the 5-year CSS rates decreased to 38.5%, 44.3%, 40.5%, and 36.9% in the high-risk groups of the four cohorts above, respectively. The significant survival difference between high-risk group (HRG) and low-risk group (LRG) indicated the precise accuracy of our risk model.

**Figure 7:**
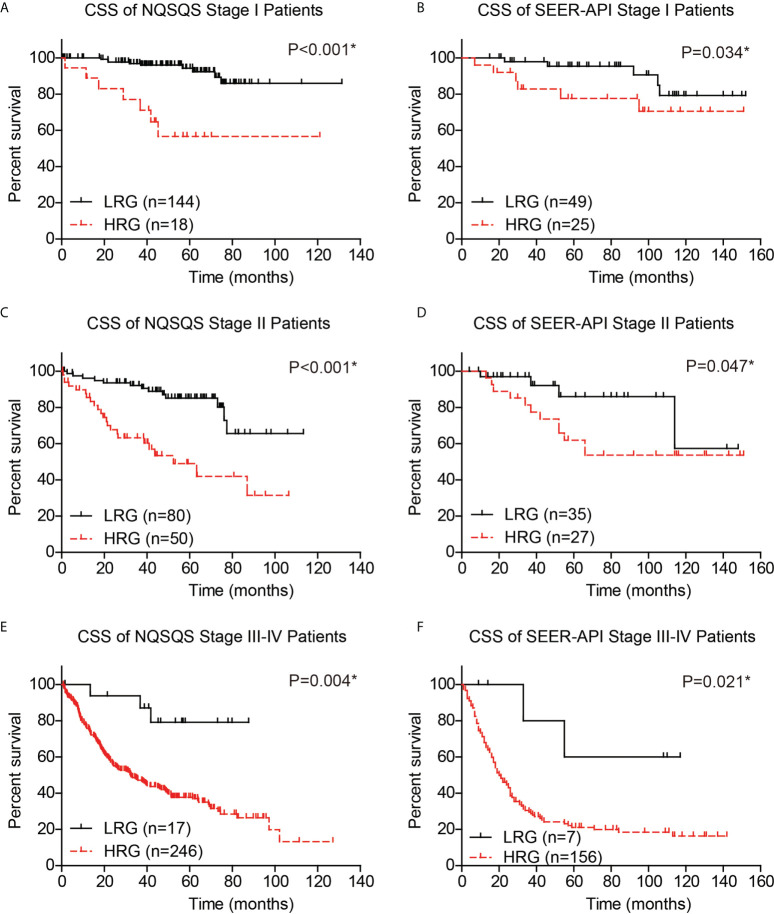
Stratification survival analyses of EOGC patients based on the risk model. The cancer-specific survival curves were plotted in the stage I NQSQS patients **(A)**, stage I SEER-API patients **(B)**, stage II NQSQS patients **(C)**, stage II SEER-API patients **(D)**, stage III-IV NQSQS patients **(E)**, and stage III-IV SEER-API patients **(F)**, respectively. *indicates P<0.05 by log-rank test.

Moreover, to validate whether our risk model can provide compensation for the TNM staging system, we independently analyzed the survival of TNM stage I, stage II, and stage III-IV EOGC patients. The 5-year CSS rate was 94.3% in low-risk stage I NQSQS patients and 56.6% in high-risk stage I NQSQS patients (P<0.001, **Figure 7A**). In the stage I SEER-API cohort, the 5-year CSS rates were 95.4% and 77.6% for low- and high-risk subgroups, respectively (P=0.034, **Figure 7B**). The 5-year CSS rate was 85.1% in low-risk stage II NQSQS patients and 49.0% in high-risk stage II NQSQS patients (P<0.001, **Figure 7C**). In the stage II SEER-API cohort, the 5-year CSS rates were 86.0% and 62.0% for low- and high-risk subgroups, respectively (P=0.047, **Figure 7D**). Similarly, a significant difference was observed in the stage III-IV patients between high- or low risk groups. The 5-year CSS rate was 79.1% in low-risk stage III-IV NQSQS patients and 37.8% in high-risk stage III-IV NQSQS patients (P=0.004, **Figure 7E**). In the stage III-IV SEER-API cohort, the 5-year CSS rates were 60.0% and 21.1% for low- and high-risk subgroups, respectively (P=0.021, **Figure 7F**). Taken together, stratification analyses highlighted the clinical significance of our model by providing additional information than TNM staging system.

### Web-based survival calculator

**Figure 8:**
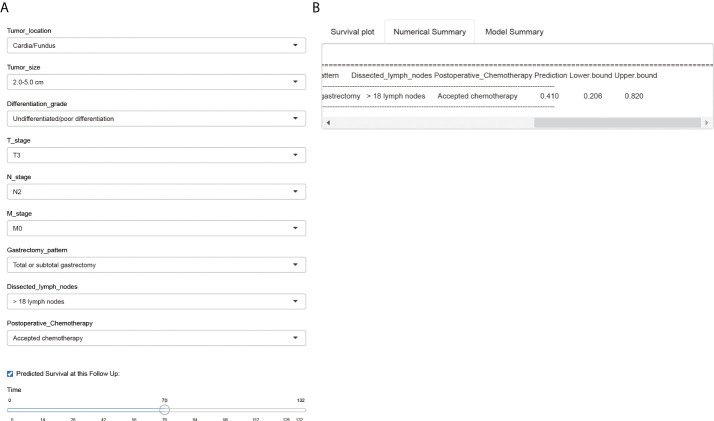
A web-based survival calculator of EOGC patients. The individual variables can be selected from the left side of the website **(A)** for cancer-specific survival prediction. The *Numerical Summary* section showed the selected variables and corresponding survival prediction results **(B)**. The *Model Summary* section showed the establishment criteria of this survival calculator.

Finally, we introduced a public-accessible web-based survival calculator to help predicted CSS of EOGC patients with personalized characteristics (https://hdliuhd.shinyapps.io/dynnomapp/). Based on the nomogram model, the survival calculator collected variables including tumor location, tumor size, differentiation grade, T stage, N stage, M stage, gastrectomy pattern, number of dissected lymph nodes, and postoperative chemotherapy (**Figure 8**). For example, if a EOGC patient was characterized with cardia tumor location, 4.0 cm tumor diameter, poor differentiation grade, T3N2M0, underwent total gastrectomy with 25 lymph nodes dissected, and accepted postoperative chemotherapy, then his predicted 5-year CSS rate was 41.0% (95% CI 20.6%-82.0%). The website is convenient and easy to use, which may hopefully help individually predict the outcome of EOGC patients.

## Discussion

According to the SEER database, the incidence of EOGC is increasing and now comprises more than 30% of all gastric malignancies in the United States (13). Consistently, Sung et al. reported that the incidence of gastric non-cardia cancer increased significantly in young adults during the past decades (14), which may correlated with the increased prevalence of autoimmune or atrophic gastritis due to usage of antibiotics and acid-suppressing drugs (15). In order to illustrate the demographic and clinicopathologic characteristics of EOGC in Asians, here we retrospectively enrolled a multi-institutional EOGC cohort in China (NQSQS cohort) and initially retrieved EOGC cases from the specific API populations in SEER database (SEER-API cohort).

EOGC was recognized to differ from conventional late-onset gastric cancers on its clinicopathological features. For example, data from the National Cancer Database of United States demonstrated that young adults with gastric adenocarcinoma are more likely to be female compared with older adults (46% vs. 35%) (16). Indeed, the female cases accounts for 44.9% and 57.9% in our NQSQS cohort and SEER-API cohort, respectively. Furthermore, EOGC patients were reported to be characterized with poorer differentiation, higher signet-ring cell tumor frequency (17), more advanced nodal metastasis and distant metastasis (18, 19). Consistently, our data showed that the signet-ring cell carcinoma comprised 33.9% cases in NQSQS cohort and 42.1% in SEER-API cohort. Meanwhile, the percentage of poor tumor differentiation or undifferentiated tumor was 74.8% in NQSQS cohort and was 86.3% in SEER-API cohort, implying its high malignant phenotype. As for the lymph node metastasis, there were 27.9% NQSQS cases and 32.8% SEER-API cases showed N3 stage. According to a Spanish study, 73.3% of EOGCs were diffuse, and up to 78.3% EOGCs were diagnosed in an advanced stage (20). Similarly, EOGC showed higher rate of Linitis plastica compared to the older group (21.7% vs. 3.4%) in a Vietnamese cohort (21). Besides the increased mortality, the outcome of EOGC was also far from satisfied. In our data, the 5-year CSS rates were 61.4% and 51.8% in NQSQS and SEER-API cohorts, respectively.

Although curative resection represents the most efficient treatment for EOGC, the postoperative prognosis differs among patients. Literature search showed that Wu et al. reported the first nomogram for young gastric cancer patients using nonmetastatic cases in SEER database as the training set (7). However, their study only enrolled patients with less than 40 years old and thus cannot fully represent EOGC cases. Besides, their nomogram may not perfectly benefit Asian populations due to different races in their training set (White or American Indian population in SEER) and validation set (Chinese population). Later Yu et al. established another nomogram to help evaluate OS and CSS of EOGC patients in SEER dataset (22). However, both the training set and validation set were selected from SEER dataset, thus lacking external validation to further confirm its clinical application and their model may be more fit to cases in United States. Similarly, Wang et al.’s prognostic nomogram was also completely based on SEER database thus possessed the same shortcoming (24). Recently, Liao et al. also reported their prognostic nomogram regarding early-onset diffuse gastric cancer (EODGC) using patients in SEER database as training set and cases from Renmin Hospital of Wuhan University as the validation set. In our opinion, the major disadvantage of Liao’s model is similar with Wu’s one, which lacks ethnicity specificity because the training cohort and validation cohort were from different races. Meanwhile, the case number in their validation set was limited (n=82) and enrolled from a single medical center, which may result in bias.

Comparing with the reported nomograms above, our model was established based on a Chinese cohort from multiple hospitals (NQSQS cohort). By dividing the NQSQS cohort into a training set and internal validation set, we introduced two nomograms and selected a better one after comparing their performance. According to the epidemiological results, gastric cancer ranks the third on the incidence of all malignancies in Japan and China (1), while it ranks 7^th^ in the United States (23), indicating racial difference between white/black populations and APIs. Considering the racial heterogeneity, we specifically retrieved the EOGC cases in API populations from SEER database for the first time, which was used as an external validation cohort. Since our model performed well in both the internal validation cohort and SEER-API external validation cohort, we safely came to the conclusion that our nomogram would be helpful for predicting the survival of Asian Pacific EOGC patients.

Furthermore, based on the risk points generated by the nomogram, we further introduced a novel risk model to distinguish high- or low-risk EOGC patients. The clinical significance of our risk model was validated by independently analyzing its predictive role in patients with different TNM stages. Finally, we provided the first online survival calculator for EOGC patients, which may help individually predicting the postoperative outcome with personalized clinicopathological characteristics.

However, our data has several limitations. Firstly, none of the patients in NQSQS cohort accepted preoperative neoadjuvant chemotherapy while this information was uncertain in SEER-API cohort. The SEER database only presents whether patient has accepted chemotherapy without specify neoadjuvant or adjuvant. We speculate that certain cases in SEER database, especially those with advanced stages, may accept preoperative chemotherapy treatment, which can help explain the fact that patients with N3 or M1 stage were more frequent in SEER-API cohort than those in NQSQS cohort. Consistently, the percentage of cases accepted chemotherapy was significantly higher in SEER-API cohort (74.2%) than that in NQSQS cohort (27.7%). Secondly, the included period range (1975 to 2016) of SEER cohort was very wide. Therefore may contain bias caused by chemotherapy difference. Thirdly, this study focused on investigating the cancer-specific survival of EOGC patients that underwent R0 resection of primary lesion, thus provided no evidence on predicting the survival of patients that lack surgical opportunity. Fourthly, our nomogram only included the clinicopathological parameters without considering the contributions of molecular biomarkers. It has been reported that EOGC was signatured with specific molecular alterations such as CDH1 germline variants (24, 25), genomic microsatellite stability (13), proteogenomic dysregulation (26), DNA damage response (27), DNA methylation (28), and alternative splicing events (29), et al. Therefore, it will be a great improvement if future studies can include molecular biomarkers in the prediction model.

## Conclusions

By summarizing the characteristics of EOGC patients in API ethnicity from SEER database and enrolled a multi-center cohort in Chinese population, we established a more precise and specific nomogram for predicting the postoperative cancer-specific survival of EOGC patients. Besides, we introduced a novel risk model and provided a web-based survival calculator for convenient clinical application.

## Data availability statement

The raw data supporting the conclusions of this article will be made available by the authors, without undue reservation.

## Ethics statement

The studies involving human participants were reviewed and approved by ethics committee of The First Affiliated Hospital of Nanjing Medical University. The patients/participants provided their written informed consent to participate in this study.

## Author contributions

HDL and ZL wrote the manuscript. QZ, QL, HZ, YW, HY, HL, XW, KL, and DW helped with data collection. XK contributed to figure illustrations. ZH, WW, LW, DZ, LY, HX, and YC helped with data analyses. YZ and ZX direct this research.

## Funding

Our study was supported by National Natural Science Foundation of China (82102723, 81871946, 82072708, 82002438, 81802406), Special Foundation for National Science and Technology Basic Research Program of China (Grant No. 2019FY101104), Fellowship of China Postdoctoral Science Foundation (2020M671392, 2020M671396), Fellowship of Jiangsu Postdoctoral Science Foundation (2020Z069, 2020Z239).

## Conflict of interest

The authors declare that the research was conducted in the absence of any commercial or financial relationships that could be construed as a potential conflict of interest.

## Publisher’s note

All claims expressed in this article are solely those of the authors and do not necessarily represent those of their affiliated organizations, or those of the publisher, the editors and the reviewers. Any product that may be evaluated in this article, or claim that may be made by its manufacturer, is not guaranteed or endorsed by the publisher.
