# Supplementary material for: Multi‑institutional development and validation of a nomogram to predict prognosis of early-onset gastric cancer patients
Source: Front Immunol. 2022 Sep 6;13:1007176. doi: 10.3389/fimmu.2022.1007176 (PMC9488636; doi:10.3389/fimmu.2022.1007176)
Supplement: Supplementary file 1 [file Table_1.docx]

Table S1. C-index of two nomogram models.

| Cohort | Model 1 | | | Model 2 | | |
| --- | --- | --- | --- | --- | --- | --- |
|  | C-index | 95% CI | P value | C-index | 95% CI | P value |
| Training set | 0.769 | 0.736-0.802 | P<0.001* | 0.798 | 0.763-0.833 | P<0.001* |
| Internal validation set | 0.732 | 0.675-0.789 | P<0.001* | 0.771 | 0.718-0.824 | P<0.001* |
| SEER-API set | 0.803 | 0.770-0.836 | P<0.001* | 0.826 | 0.793-0.859 | P<0.001* |
