# Supplementary material for: Multi‑institutional development and validation of a nomogram to predict prognosis of early-onset gastric cancer patients
Source: Front Immunol. 2022 Sep 6;13:1007176. doi: 10.3389/fimmu.2022.1007176 (PMC9488636; doi:10.3389/fimmu.2022.1007176)

**Figure S1. Calibration curves of model 1 nomogram.**

Calibration curves of the nomogram in the training set (A), internal validation set (B), and SEER-API external validation set (C) were plotted based on 1-, 3-, and 5-year CSS, respectively. The X-axis represents the model‑predicted survival, and the Y‑axis represents actual survival. The bar represents 95% CI measured by Kaplan–Meier analysis, and the dotted line represents the ideal reference line.


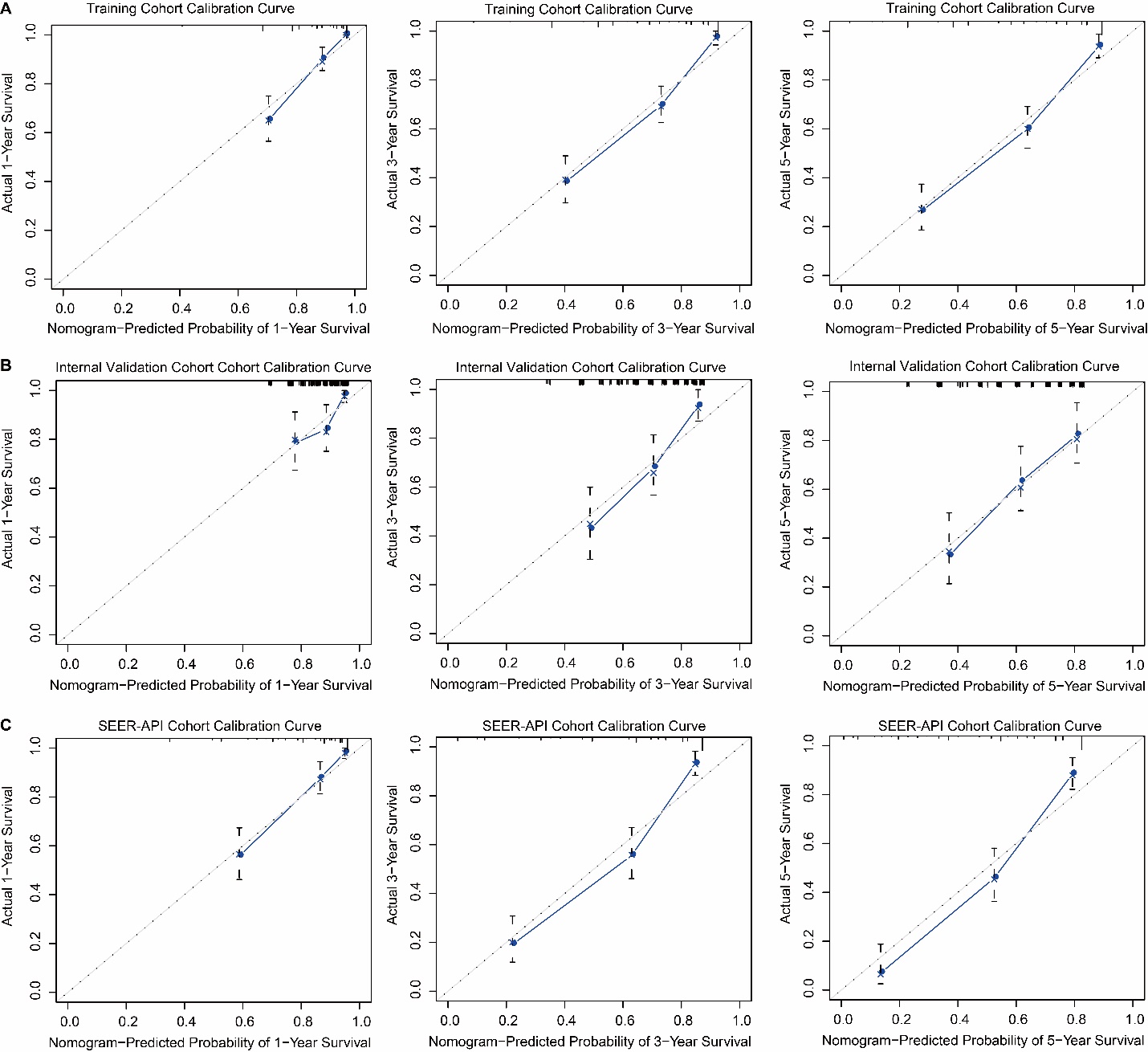


**Figure S2: X-tile analysis of survival data from the training set.**

X-tile plot of the training set is displayed in the (a). The optimal cutoff value marked by the black circle in the Figure 1a is shown by a histogram of the entire cohort (b), and a Kaplan–Meier plot (c).

The figure shows the optimal cutoff points for distinguishing high-risk (points >188) and low-risk (points ≤ 188) EOGC patients.


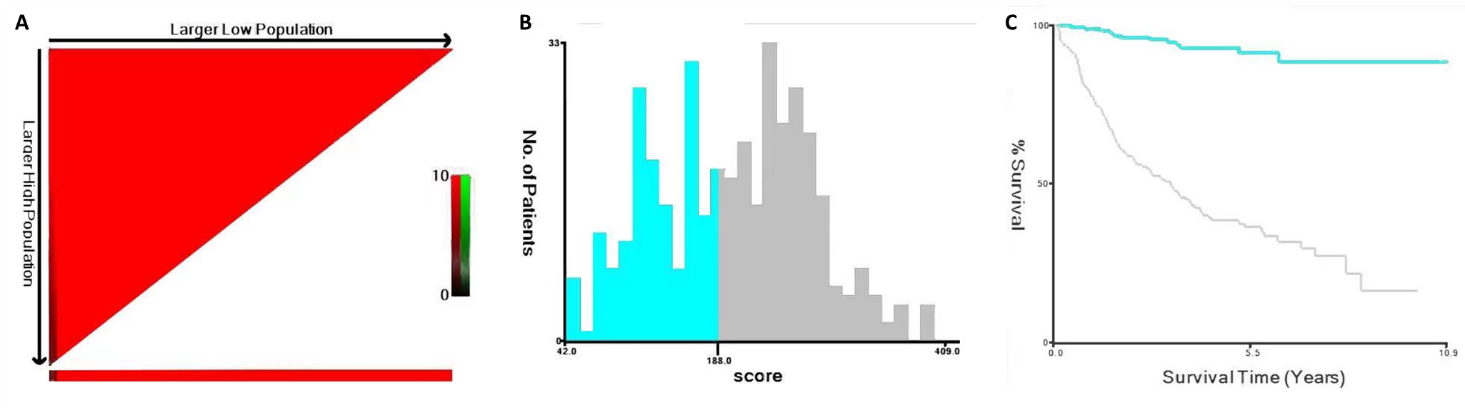

Supplement: Supplementary file 2 [file DataSheet_1.docx]
